# Temporal Control and Hand Movement Efficiency in Skilled Music Performance

**DOI:** 10.1371/journal.pone.0050901

**Published:** 2013-01-03

**Authors:** Werner Goebl, Caroline Palmer

**Affiliations:** 1 Institute of Music Acoustics, University of Music and Performing Arts Vienna, Vienna, Austria; 2 Department of Psychology, McGill University, Montreal, Quebec, Canada; McMaster University, Canada

## Abstract

Skilled piano performance requires considerable movement control to accomplish the high levels of timing and force precision common among professional musicians, who acquire piano technique over decades of practice. Finger movement efficiency in particular is an important factor when pianists perform at very fast tempi. We document the finger movement kinematics of highly skilled pianists as they performed a five-finger melody at very fast tempi. A three-dimensional motion-capture system tracked the movements of finger joints, the hand, and the forearm of twelve pianists who performed on a digital piano at successively faster tempi (7–16 tones/s) until they decided to stop. Joint angle trajectories computed for all adjacent finger phalanges, the hand, and the forearm (wrist angle) indicated that the metacarpophalangeal joint contributed most to the vertical fingertip motion while the proximal and distal interphalangeal joints moved slightly opposite to the movement goal (finger extension). An efficiency measure of the combined finger joint angles corresponded to the temporal accuracy and precision of the pianists’ performances: Pianists with more efficient keystroke movements showed higher precision in timing and force measures. Keystroke efficiency and individual joint contributions remained stable across tempo conditions. Individual differences among pianists supported the view that keystroke efficiency is required for successful fast performance.

## Introduction

Skilled piano performance is a highly refined and demanding human skill and requires enormous control of movement to accomplish the precise timing required of Western tonal music performance. Pianists practice thousands of hours over more than a decade to achieve a skilled technical (and musical) level [1]. Among the most difficult of tasks is performing fast and loud [2], due to increased force production in short time periods. This paper examines pianists’ finger and hand movements during performances of a simple melody at different rates, and compares movement efficiency with temporal precision and accuracy of the performances.

Since Bernstein and Popova’s seminal study [3] of pianists’ repeated octave strikes produced with different tempo and force instructions [4], there have been several scientific investigations of arm and hand motion in piano performance. Furuya and Kinoshita [5] employed an octave-striking task (similar to Bernstein and Popova [3]) and a 2D motion capture system to compare repeated strikes of experts and novices; they found more consistent and efficient movements in experts than in novices. Using another task (a tremolo spanning the interval of a sixth) and 3D motion capture, Furuya and colleagues [6] found similar results: Professionals tended to perform with larger degrees of freedom, use less muscular force, and generated the motion from more proximal parts (wrist rotation) of the movement chain than did novices. However, the tasks used in the above-mentioned studies represent quite advanced and highly pianistic movement patterns specific to certain musical styles of Western tonal music [7] and, thus, may not generalize to more fundamental movements required of piano performance. Fast octave repetitions were not introduced into the piano repertoire widely until the compositions of Franz Liszt [8], whereas finger movements required to perform melodic patterns with small successive pitch intervals, such as those found in musical scales, are common in Western music [9] and are part of every young pianist’s practice regime [10]. The present study investigates finger and hand movements during performances of a simple melody that requires scalar five-finger movements, which are fundamental to the technical requirements of the standard piano repertoire [11].

Several studies have investigated effects of performance tempo on pianists’ fingertip movement properties during performance. As the tempo became faster, pianists raised their fingers farther above the keyboard [12], the fingertip velocities towards the key increased [12], [13], and the kinematic landmarks in the fingertip trajectories, such as maximum finger height or finger-key contact [14], extended over a larger portion of an inter-onset interval [13]. Furthermore, there is evidence to suggest that proprioceptive feedback from the fingertip can counterbalance a speed-accuracy tradeoff underlying the tendency toward larger temporal variability at faster tempi [6], [12], [14], [15]. Furuya and colleagues [16], [17] used a data glove to examine movement covariation between finger joints of pianists who performed musical excerpts from the Classical-Romantic piano repertoire at two tempi (a normal tempo and an “as fast as possible” tempo). They reported no change with tempo in the observed covariance of finger joint movements, and attributed it as emerging from extensive piano practice [17]. However, their data included only two joints of each finger, and only two tempi, one of which differed across pianists. The present study examines the influence of 10 levels of performance tempo on all joint motion from forearm to fingertip during pianists’ keystrokes.

Pianists’ methods of finger technique (patterns of finger movement that generate sound) are considered a fundamental building block of piano performance [11], and may involve different movements than octave strikes and tremolo movements addressed in the above-mentioned studies. The anatomical and physiological foundations of a finger-stroke (downward finger movement toward piano keys to create one tone) are documented as very complex [13], [18]. The fingertips are flexed by the deep flexor muscles in the forearm, the middle phalanx by the superficial flexor muscles, both via tendons that run from the forearm through hand ligaments and tendon sheaths to the fingers (extrinsic muscles of the hand). The proximal phalanx of the fingers is moved primarily by the intrinsic muscles of the hand (lumbrical and interosseus muscles). As the interosseus muscles move the fingers sideways (the dorsal interosseus spread the fingers, the palmar draw them together) [18], and the lumbrical muscles flex the knuckle (the metacarpal-phalangeal joint, MCP). However, the lumbricals are attached to the deep flexor tendon at the hand end and to the extensor tendon expansion on the finger end. Thus, while they flex the knuckle, they also straighten the fingers by pulling the extensor tendon expansion ([18], p. 57). The deep flexor muscles are strong but slow, and exhibit low independence between the fingers [19], while the lumbricals are quite the opposite: weak, fast, and independent [20].

There has been little attempt to incorporate anatomical and physiological knowledge of finger movements in scientific explanations of piano technique [21]. One exception is Otto Ortmann [22] who accomplished precise recordings of different types of finger keystrokes with self-designed mechanical equipment. He proposed a distinction between a flat-finger stroke and a curved-finger stroke. The flat-finger stroke features a straight finger that is moved exclusively by the knuckle (MCP), while the other finger joints (the distal and the proximal interphalangeal joints, DIP and PIP) remain immobile ([22], p. 221). The flat-finger stroke allows fast finger speeds but lower forces according to the lever principle [7]. The curved-finger stroke features flexed finger joints, while the MCP is extended (so that the nail joint is completely vertical, see [22], p. 221). During a pianist’s keystroke, the finger extends as the knuckle flexes, the nail joint remaining almost vertical throughout. This type of keystroke allows more forceful playing than the flat-finger stroke. Thus, flat fingers should be preferred for fast scale playing, while curved finger more for loud and forceful playing [11]. Current scientifically informed piano educators recommend finger strokes somewhere in-between Ortmann’s extremes of curved and straight-finger strokes [23]. Richard Beauchamp describes a “slightly curved” finger that is “allowed to unbend” (“not consciously straightened”) during a keystroke to be an optimal configuration of an independent finger technique [24], [25].

We examined the finger and wrist movements of highly skilled pianists who performed a simple five-finger exercise with the right hand at different tempi. We analyzed the joint angles between adjacent segments of the fingers, the hand, and the forearm to identify the contribution of each joint rotation to particular keystroke movements (defined as the movement of a fingertip toward a piano key prior to its depression). We focused on the effects of tempo on the particular finger, wrist, and arm movement properties and on individual differences between pianists. Also, we introduced a keystroke efficiency measure that quantified the extent to which the individual joints in a movement chain work together toward the keystroke movement goal. This keystroke efficiency measure was compared with auditory measures of the music performance outcome (temporal accuracy, precision; loudness precision) in order to identify movement properties that might support successful music performance.

## Methods

### Ethics Statement

All experimental protocols were reviewed and approved by the Research Ethics Review Committee of McGill University, and written informed consent was obtained from all participants.

### Participants

Twelve skilled pianists (defined as those who have received piano instruction for at least 10 years) from the Montreal area participated in this study. They were 20–33 years old (mean = 27.7 years) and had 10–25 years of piano lessons (mean = 18.7 years; one participant was 61 years old with 40 years of lessons); they were all trained to perform the Classical–Romantic piano repertoire. They received a nominal fee for their participation.

### Design and Procedure

One isochronous melody was created for the right hand that could be repeated (cycled) continuously and was easy to perform at fast tempi. The tempo conditions were 7.0, 8.4, 9.6, 10.7, 11.7, 12.3, 13.3, 14.1, 15.0, and 16.0 tones per second (or 143, 119, 104, 94, 85, 82, 75, 71, 66, and 62 ms inter-onset intervals, respectively). The experiment employed a synchronization-continuation paradigm using a metronome to signal the tempo at the beginning of each trial, and each pianist performed the melody at different tempi, starting with the slowest tempo condition. On each trial, the pianists first synchronized their performance with the metronome; after the first cycle of the melody, the metronome stopped and the pianists continued the tempo for five repetitions. Three trials were performed at each tempo before the metronome tempo was increased to the value of the next tempo condition, until the pianists decided to stop (they were pushed to the limits). At the end of the experiment, which lasted approximately 1 hour the pianists completed a musical background questionnaire.

### Equipment

**Figure 1:**
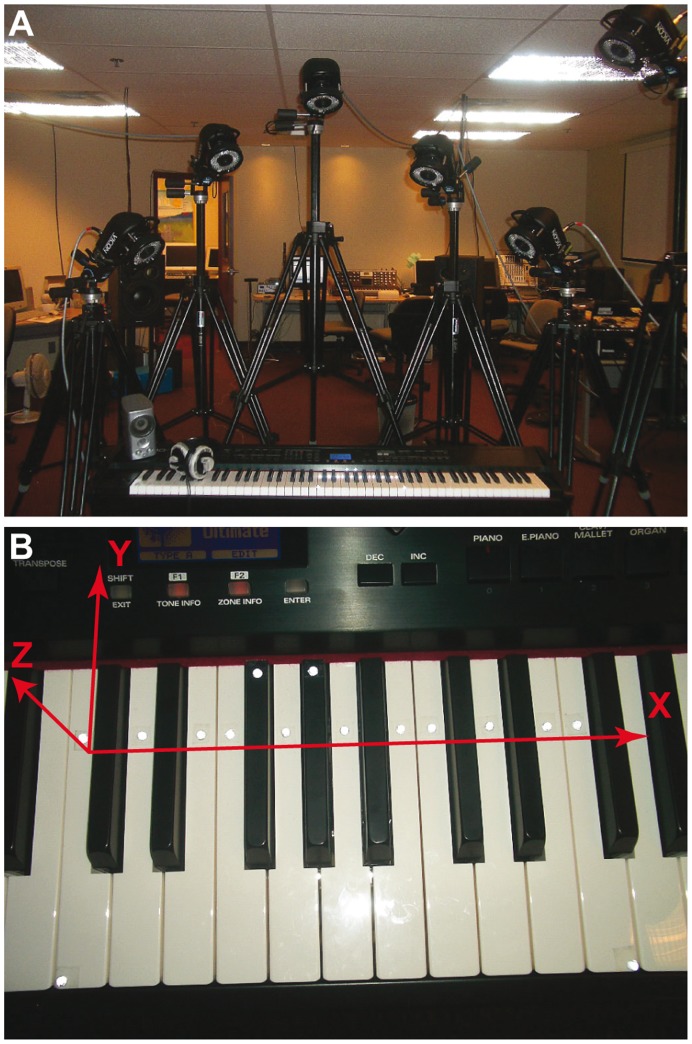
Motion capture setup and marker placement. The motion capture setup involved 6 infrared cameras arranged around the digital piano (top). Fifteen markers were placement on the keyboard with the dimensions sketched in red (bottom).

**Figure 2:**
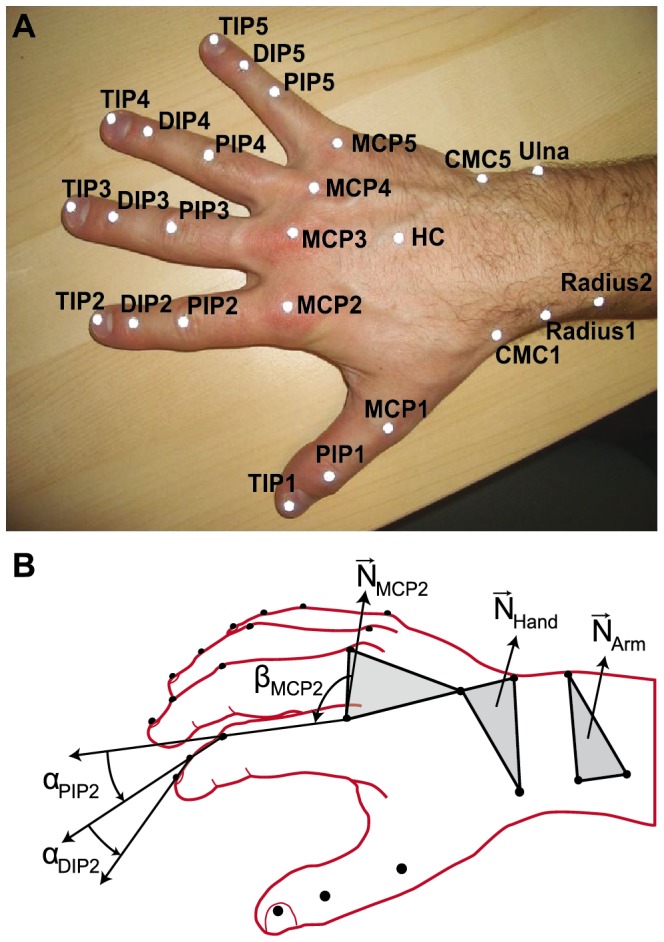
Hand markers and joint angles. Twenty-five markers were placed on the fingers, hand, and forearm with corresponding labeling (top). Schematic of the calculation of individual joint angles: surface normals and angles (bottom).

A passive motion capture system (Vicon 460 by Vicon, Los Angeles, CA, USA) equipped with six infrared cameras (MCam2) tracked the movements of 4-mm reflective markers at a sampling rate of 250 frames/s. Fifteen markers on the piano keys monitored the motion of the keys and were used to determine the plane of the keyboard, shown in Figure 1. Another 25 markers were glued on pianists’ finger joints, hand, and wrist to track the pianists’ movements during the performances. The exact marker labels and placement on the hands is shown in Figure 2.

The pianists performed on a MIDI digital piano (RD-700SX by Roland Corporation, Los Angeles, CA, USA). The pianists were allowed to adjust the height of the piano stool until they felt comfortable at the piano. An electronic metronome (Dr. Beat DB-88) was placed on the piano in front of the pianists to indicate the nominal performance tempo. Pianists heard the piano via closed headphones (AKG K271) plugged directly into the digital piano; they heard the metronome from a separate speaker placed on the digital piano. The metronome signal and the MIDI data were recorded on a personal computer with a digital soundcard (MOTU 828mkII sampling at 44.1 kHz) using Cubase software. The metronome signal was also recorded on the analog input of the Vicon system (a 32-channel Mezzanine card sampling at 10 kHz) and the motion and keyboard data were aligned, based on the metronome signal; the synchronization error of this method was less than 1 ms. Subsequent timing analyses of the performances were based on the MIDI data.

### Data Analysis

The three-dimensional motion data was rotated so that the height dimension (marked Z in Figure 1) was orthogonal to the keyboard plane (right/left and front/back, marked X/Y in Figure 1). The trajectories of the 25 finger-tip markers were converted with functional data analysis techniques using order-6 b-splines fit with knots placed every five data points and smoothed with a roughness penalty *λ = *10^–18^ on the fourth derivative of X/Y/Z position, smoothing the second derivative (acceleration) [26]. Kinematic landmarks were extracted from each finger movement toward the keys in the height dimension above the keyboard plane: the maximum finger height (mxH, interpreted as the beginning of the finger movement) and the key-bottom landmark (KB, when the finger is stopped by the keybed) [12], [13], [14], [27]. Additional 3 knots were placed at the acceleration peaks at the KB landmarks to preserve these acceleration extremes across the functional data smoothing.

Joint angles were computed for all adjacent phalanges of the fingers, the wrist and the forearm. Starting from the wrist, we computed the wrist angle (between forearm and the metacarpals), wrist rotation (the degree of pronation/supination relative to the keyboard plane), and for each of the fingers, three joint angles (metacarpophalangeal joint MCP, proximal inter-phalangeal joint PIP, and distal inter-phalangeal joint DIP). Finger 1 (the thumb) was excluded from analysis because its angles move differently than the other fingers (index finger, middle finger, ring finger and pinkie) and the melody required fewer thumb movements.

The precise angle definitions were as follows: The wrist angle was computed as the angle between a plane defined by radius1, radius2, and ulna (represented by normal *N_Arm_*, see Figure 2) and a plane defined by the carpometacarpal markers CMC1 and CMC5, and the center marker of the back of the hand (HC, represented by normal *N_Hand_*), such that a negative value refers to wrist extension and positive values to wrist flexion [cf., 28].

The wrist rotation is computed as the angle of the line CMC1 to CMC5 relative to the horizon (defined by the keyboard plane), such that positive values reflect supination or rotation toward the 5^th^ finger, while negative values reflect pronation or rotation towards the 1^st^ finger.

The MCP angle *α_MCP_* was defined as the angle between the base segment (from MCP to PIP) and the hand back plane (represented by its normal vector *N_MCP_*). The hand back planes were different for each finger: For finger 2, the hand back plane was defined by MCP2, MCP3, and HC; for finger 3 and 4, MCP3, MCP4, and HC, and for finger 5, MCP4, MCP5, and HC.

For each hand back plane, its surface normal (orthogonal to the plane) was computed. For example, the surface normal of MCP2 is:
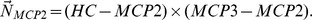



The MCP angle α is computed using the surface normal as follows:
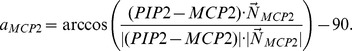



Figure 2 bottom shows *β_MCP_* defined as: 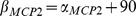. A positive value of *α_MCP_* denotes flexion of the base segment; a negative denotes extension. This method for computing the MCP angle does not take abduction or adduction movements into account, which supposedly play a minor role in the present task.

The PIP angles were computed using PIP normals that retain the orientation of the MCP normals:
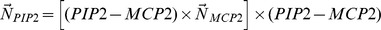



The PIP2 angle is computed between the middle segment and the PIP normal of finger 2, thus:
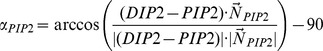



Again, a positive *α_PIP_* value denotes flexion; a negative value denotes. The DIP angles were calculated the same as for the PIP angles.

Behavioral measures obtained for the sounded piano performances included accuracy and precision of timing, and precision of keystroke velocities. Specifically, we examined the timing accuracy with the signed timing error [(observed IOI – expected IOI)/expected IOI] and the timing precision with the coefficient of variation, CV, of the inter-onset intervals (SD/mean) [29]. In addition, we examined the precision of keystroke intensities with the coefficient of variation, CV, of the tone velocities (SD/mean key velocity).

## Results

**Figure 3:**
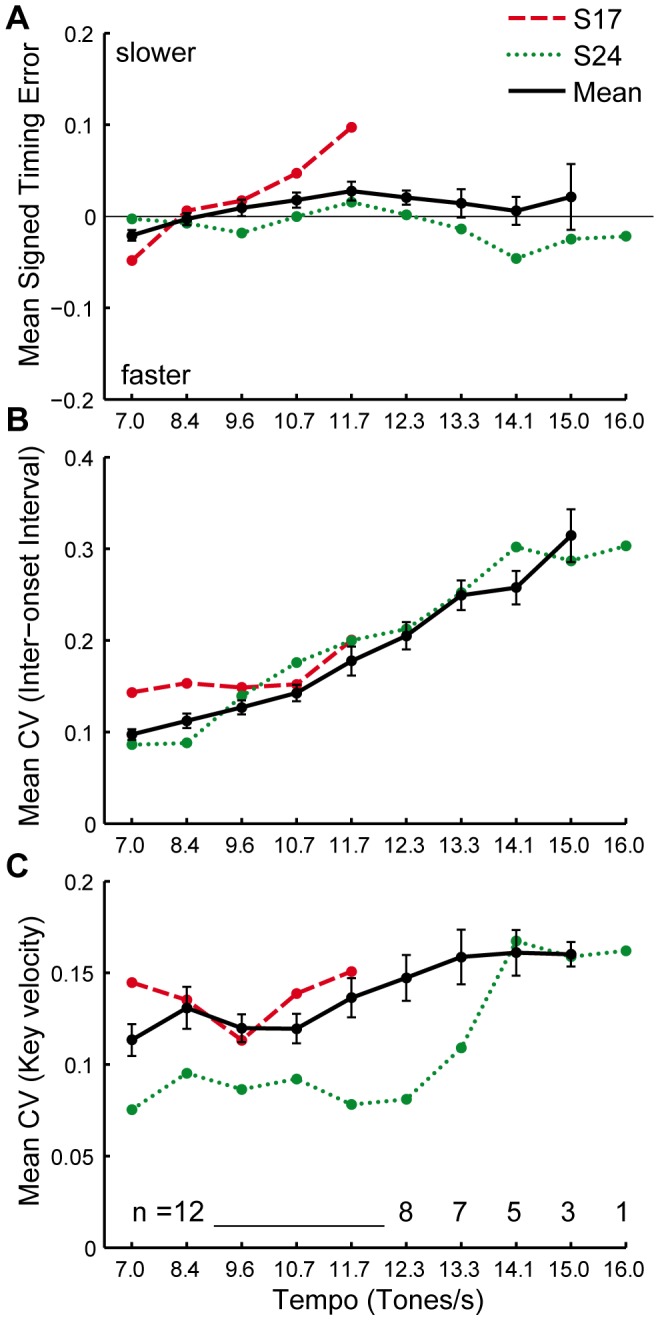
Measures of performance quality. Means and standard errors of the means for all 12 pianists, the “slow” pianist (S17) and the “fast” pianist (S24) shown separately, for (A) signed timing error (%), (B) the mean coefficient of variance (SD/mean) of the inter-onset intervals (IOI in ms), and (C) the mean CV of key velocity (in MIDI units).

All pianists were able to perform the melodies at the five slowest tempo conditions (up to 11.7 tones/s); decreasing numbers of pianists were able to perform at the successively faster tempi (as shown in Figure 3). One pianist, S24, was able to perform in all tempo conditions including 16.0 tones per second (which corresponds to a metronome marking faster than the fastest pieces in the Western tonal repertoire); we refer to this pianist as the “fast pianist”. Another pianist, S17, accomplished only the five slowest tempo conditions and practiced the fewest number of hours in the previous week; we refer to this pianist as the “slow pianist” for comparison.

### Acoustic Measures of Performance

The timing of tone onsets (measured by the digital piano) and tone intensity (measured by MIDI key velocity) are the two main acoustic parameters that pianists can manipulate. Figure 3 shows the signed timing error, the coefficient of variation for inter-onset intervals, and the coefficient of variation of the tone velocities for the group of 12 pianists and for the “slow” and “fast” individuals, S17 and S24. Repeated-measures analyses of variance on each of the measures indicated significant increases in timing error (Figure 3A) and in timing variability (Figure 3B) across the first 5 tempo conditions (those in which all 12 pianists performed), but not in loudness variability (Figure 3C): the main effect of tempo was significant for signed timing error, *F*(4,44) = 10.35, *p*<.001, and for CV of IOIs, *F*(4,44) = 15.70, *p*<.001, but not for CV of key velocities, *F*(4,44) = 1.62, *p* = .187). As shown in Figure 3A, S17 performed faster than the metronome at the first tempo condition, but increasingly slower at faster tempo conditions, while S24 performed more accurately than other pianists throughout all conditions. S17 was also more variable in timing at slow tempi than S24 (Figure 3B), and generally more variable in tone intensities than S24 (Figure 3C).

### Finger and Wrist Joint Angles

**Figure 4:**
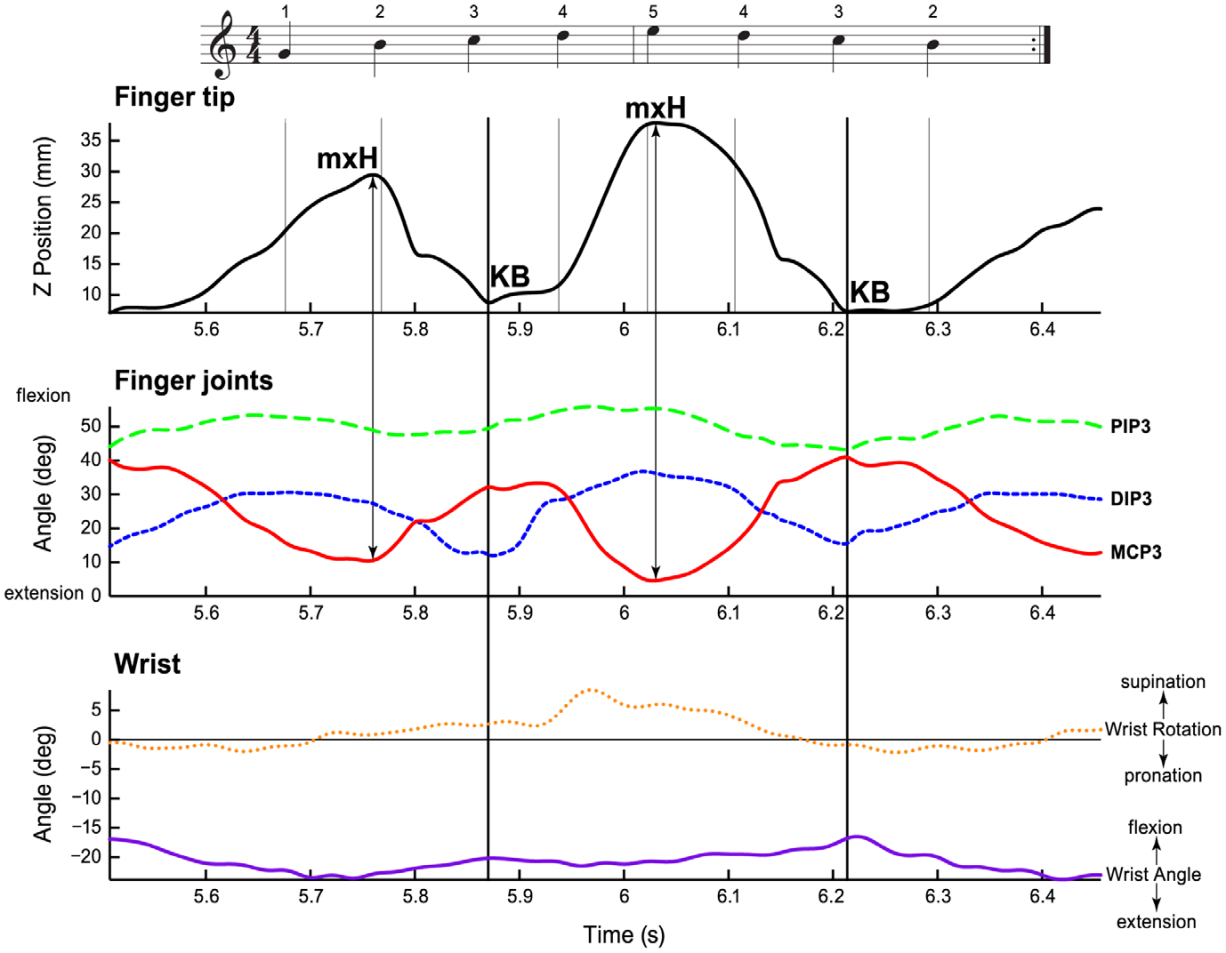
Exemplary joint angle data. One cycle of the melody performed by S24 at 11.7 tones per second. Top panel shows the fingertip trajectory for the middle finger (finger 3); the middle panel shows the finger joint angle trajectories (in degrees) for the metacarpophalangeal joint, MCP (red line), the distal interphalangeal joint, DIP (dotted blue line), and the proximal interphalangeal joint, PIP (dashed, green line); the bottom panel shows wrist angle (purple solid) and wrist rotation trajectories (orange dotted). Thin vertical lines in the top panel indicate the tone onsets (KBs) of the 8 tones of one cycle of the melody as printed in music notation above the figure.

We examined the angle trajectories and the finger tip position trajectories within each melody cycle (across 8 keystrokes). Figure 4 shows S24’s fingertip trajectory for Finger 3 (top panel), the finger 3 angles (DIP, PIP, and MCP, middle panel), and the wrist angle and rotation (bottom panel) across one melody cycle. Finger 3 strikes a piano key twice during each melody cycle, marked Key Bottom (KB). The maximum height (mxH) and the KB landmarks are reflected in the finger joint angles, particularly in the MCP. In contrast to the finger joint angles, the wrist angle (up/down movement of the hand) and the range of wrist rotation (supination/pronation) stayed relatively constant across the melody cycle. The average range of angles within a cycle was larger for the finger joints (DIP: 30.06°, PIP: 23.73°, MCP: 33.98°) than for the wrist measures (Wrist angle: 12.63° and wrist rotation: 9.39°). The mean wrist angle across pianists was –10.81° (slight extension) corresponding roughly with the recommendations by Tubiana and colleagues as an optimal wrist position for MCP function [28].

**Figure 5:**
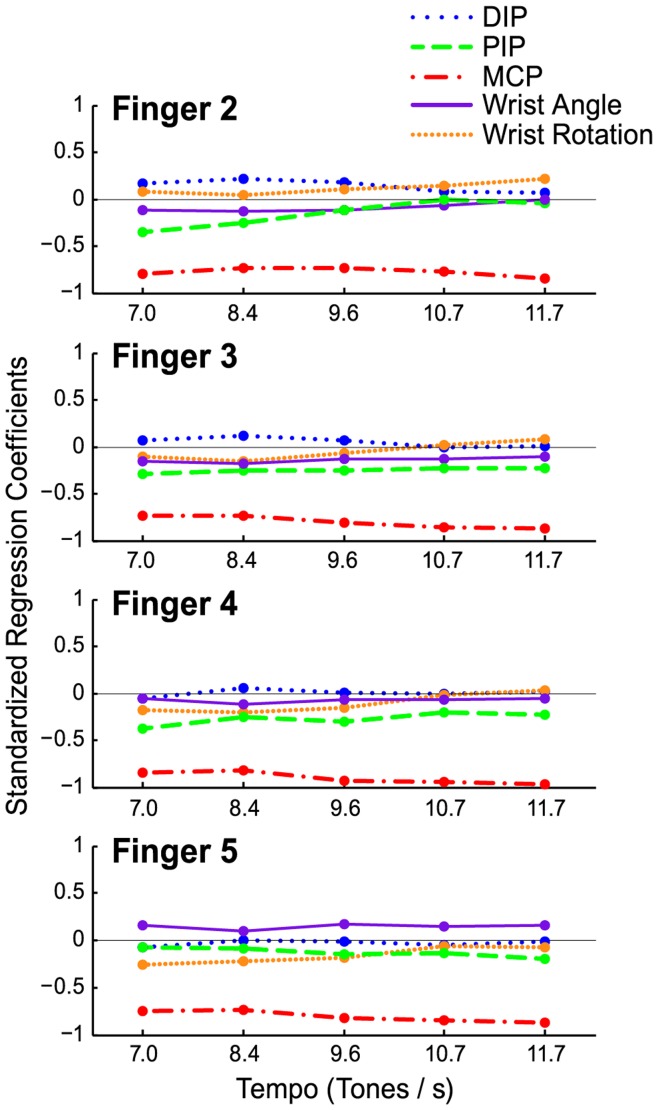
Fingertip position predicted by joint angles. Standardized regression coefficients of multiple regression models predicting each fingertip trajectory (4) from the five joint angle trajectories within tempo condition, plotted by finger and tempo condition.

Next, we examined interdependencies among the wrist and finger angle trajectories and the fingertip movements on a per-cycle basis using multiple regression models. The fingertip position (Figure 4, top panel) was predicted from each of the five angle measures shown in Figure 4 (lower two panels) for each pianist’s fingers, cycles, and tempo conditions. The standardized regression coefficients from the 20 multiple regression analyses, predicting fingertip position (z) from the five joint angles are shown in Figure 5 by tempo condition and finger. The multiple regression coefficient, *R*, from the fits to each finger was highly significant (multiple *R*’s >.90, *p*<.001) as were each of the standardized beta coefficients for each tempo (*p*’s <.001; the only exception was finger 3′s wrist rotation at tempo 9.6 tones per second with a *p*-value of 0.367). Semi-partial correlation coefficients for each joint angle confirmed that MCP contributed most to fingertip position.

### Joint Contributions to Keystrokes

**Figure 6:**
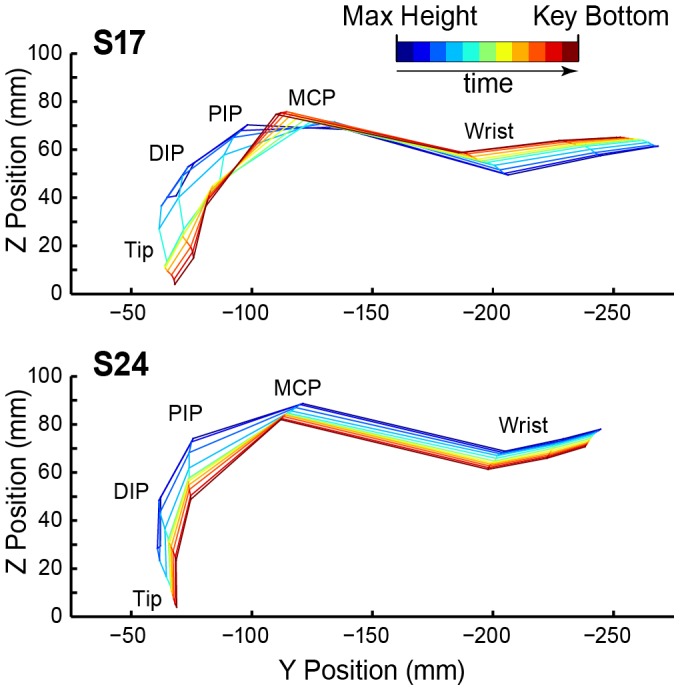
Two keystrokes produced by different pianists. Stick figure display of a finger-3 keystroke for subjects 17 (top) and 24 (bottom) viewed from a side perspective. The individual lines represent equally spaced time slices from time of maximum height (blue) to key bottom (dark red).

To test the relationship between finger movements and acoustic goals of timing and tone intensities, we next examined the interdependencies of the finger joint angles during each keystroke. The keystroke movement trajectories that generate sound in piano performance have been defined from the maximum height (mxH, see Figure 4 top panel) of the fingertip prior to a produced tone to the arrival of the fingertip at key bottom (KB) [12], [27]. Figure 6 demonstrates a keystroke from a side perspective for the “slow” pianist S17 (top) and the “fast” pianist S24 (bottom), from the time of mxH to KB for Finger 3 movement toward a key. Subject 17’s movement shows increased flexion of MCP joint and bending of DIP inwards (extension) toward the end of the keystroke, whereas S24’s movement shows reduced MCP flexion and continued DIP flexion throughout the entire movement.

A video of S17 and S24 performing at tempo 5 verifies the difference in joint angle usage (Video S1). The video shows hand reconstructions driven by the smoothed marker data of S17 on the left and S24 on the right. The spheres in the foreground are markers attached to the piano keys. The original recordings were slowed down by a factor of 5.4 and 5.8, respectively (as S17 performed at a slightly slower rate than S24), to match each other visually.

**Figure 7:**
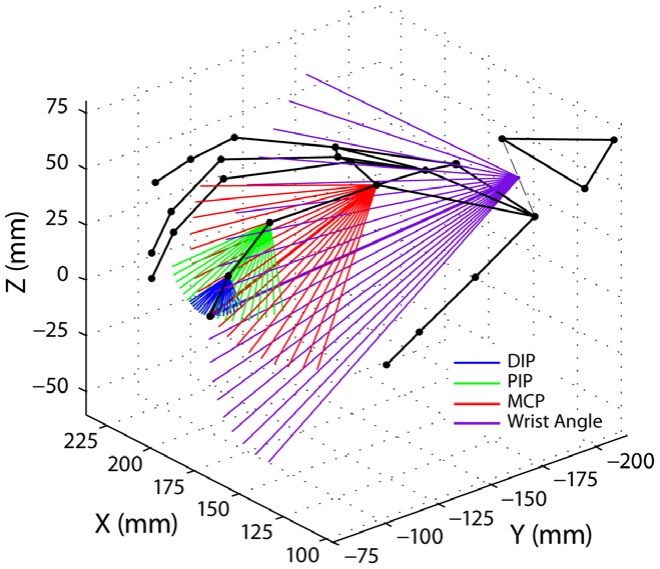
Joint angle simulation. Individual joint angle contributions to simulated fingertip motion from maximum height (mxH in Figure 1) toward key bottom. Black dots indicate the marker position at time of maximum height above keyboard. For each of the four joint angles of the index finger (DIP blue, PIP green, MCP red, WRIST purple), we show the simulated fingertip movement for joint angles ranging from –45° (extension) to 45° (flexion) in steps of 5° with the assumption that only that particular joint contributes to the movement (all other joint angles unchanged).

To examine how each finger joint contributes to the final movement of the fingertip, we conducted an analysis that takes into account the fundamentals of lever mechanics of the fingers and the hand. To demonstrate this, we show in Figure 7 simulated fingertip movements for exemplary joint angles of finger 2 ranging from –45° (extension) to 45° (flexion) in steps of 5° under the simplifying assumption that only one particular joint angle: DIP (blue), PIP (green), MCP (red), or WRIST ANGLE (purple), contributed exclusively to that final fingertip motion, while the other, more distal joints were kept constant at the angles measured at the time that finger 2 reached maximum height before a tone onset. The middle (thicker) of the 19 lines for each joint represents an angular change of 0° (the observed joint angle) and connects to the observed fingertip position. This simulation exemplifies that the same amount of angular change in the four joints gives rise to very different fingertip movements, depending on the orientation of the joint rotation axis and on the length of the lever from the joint to the fingertip (Figure 7).

To quantify each finger joint’s contribution during a particular keystroke, we computed the vertical distance (Z plane) that the fingertip travelled from time of Maximum Height (mxH) to time of Key Bottom (KB), labeled *ΔTIP* (in mm), as well as the change in each joint’s angle (DIP, PIP, MCP, and Wrist, in °) over the same time period. From each joint angle change, we calculated an *estimated fingertip position* at time of KB under the assumption that only that particular joint moved and the other joints remained immobile at the angle configuration measured at the time of maximum height. To generate this estimate, we rotate the line from the corresponding joint to the fingertip (see Figure 7) by the amount of change in joint angle, thus avoiding the more complex estimate based on individual joint segments and each of their relative angles. We next computed the vertical distance (in mm) from the mxH fingertip position to the estimated KB fingertip position due to each individual joint, called the estimated vertical distance (*estΔTIP_DIP_, estΔTIP_PIP_, estΔTIP_MCP_, estΔTIP_WRIST_*). We report these estimated vertical distances as a proportion of the actual vertical distances of the fingertip (*ΔTIP*).

**Figure 8:**
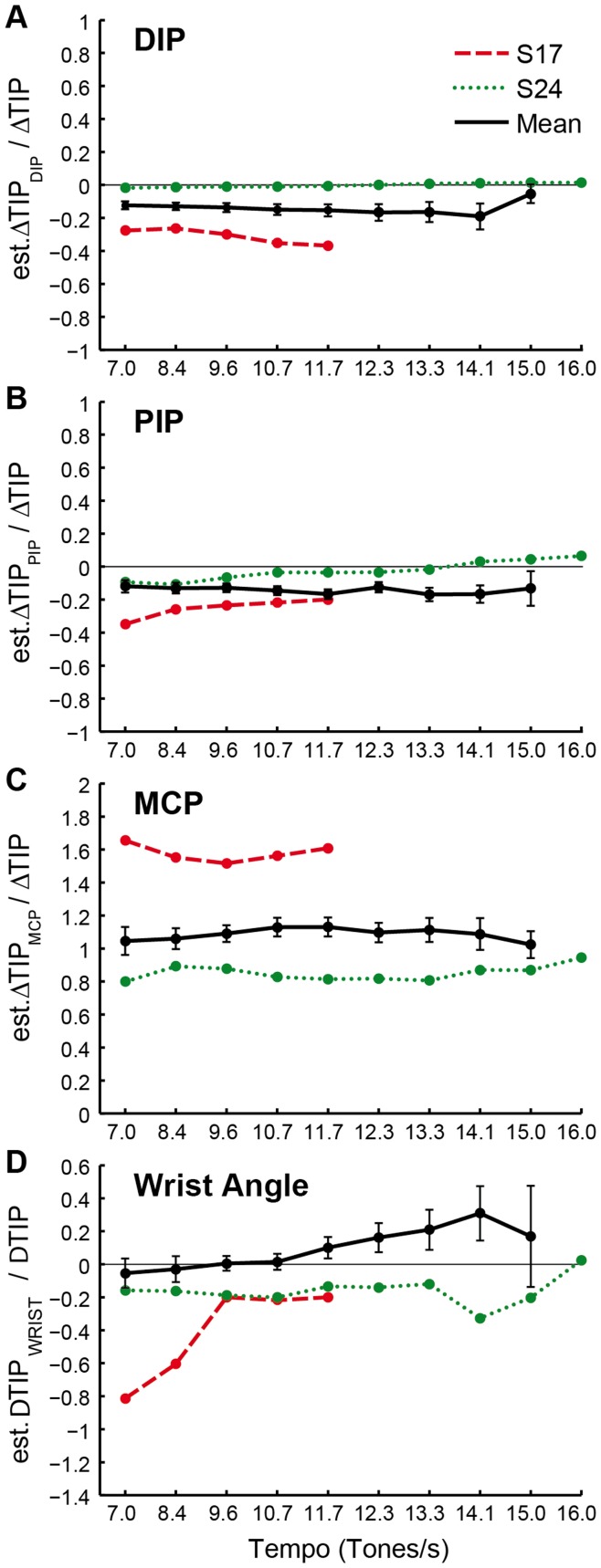
Individual joint contributions to fingertip motion. Individual joint contributions (estΔTIP_DIP_, estΔTIP_PIP_, estΔTIP_MCP_, estΔTIP_WRIST_) to vertical fingertip motion for finger 2 plotted as a proportion of the actual vertical fingertip movement from time of maximum height to key bottom (ΔTIP) for all pianists (mean) and the two individuals S17 and S24. Error bars indicate standard error of the mean.

Figure 8 shows these proportions for finger 2 across all pianists and the two particular individuals, S17 and S24, for each of the four joints DIP, PIP, MCP, and WRIST. Positive values denote joint contributions toward the movement goal, while negative values signify joint contributions that work against the movement goal; values around 1 imply that a particular joint’s movement alone could account for the total movement of the fingertip (ΔTIP). The patterns in Figure 8 suggest that, on average, MCP is the only joint generating movement in the direction of the movement goal, while the small negative values of PIP, DIP, and Wrist indicate smaller movement opposite in direction to that of the fingertip. Separate 2-way repeated-measures ANOVAs on each of the four joint angle proportions shown in Figure 8 (DIP, PIP, MCP, WRIST) by finger (2–5) and tempo yielded differences among fingers, but no main effects of tempo or any interactions. This suggests that each finger joint does not change its relative contributions to the fingertip movements across tempi.

Examination of the two individuals’ data suggests that S17’s MCP would have generated a fingertip movement 1.6 times greater than what the actual fingertip movement, if the other joints had not moved at all. But they moved opposite to the downward direction of the fingertip goal, so the MCP had to work hard to achieve the keystroke. In contrast, S24’s MCP contributed about 0.9 times the actual fingertip movement, while PIP moved only slightly away and DIP did not contribute at all. The stick figure display of S17’s fingertip movement (see Figure 6) shows a considerable extension of the DIP joint, particularly after the finger made contact with the key surface. This is referred to in the literature as “breaking-in of the nail joint” ([30], p. 225). This breaking-in is proposed to be due to a lack of tendonous support of the DIP joint by the deep flexors and is generally considered detrimental to tone control and precision, as the movement initiated by the MCP is damped and not transmitted directly to the key [11], [21]. This viewpoint is also supported by S17’s way of striking the keys, which shows this breaking-in particularly in fingers 2–4 and may be one of the reasons for S17’s extreme operation of the MCP joint.

### Keystroke Efficiency

**Figure 9:**
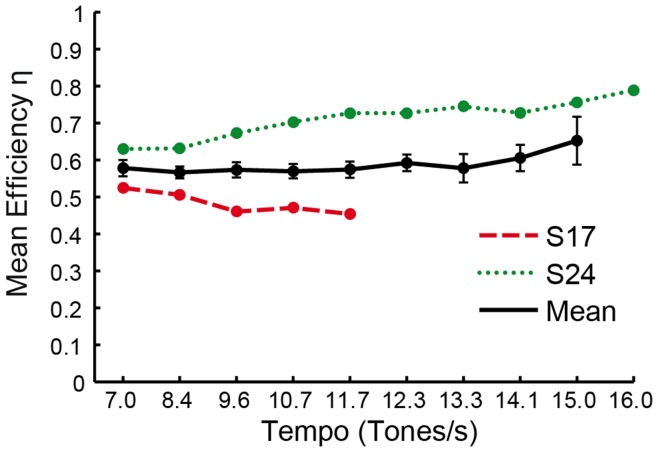
Keystroke efficiency. Mean efficiency η by tempo condition for all pianists and two individuals S17 and S24. Error bars indicate standard error of the mean.

Finally, we combine the estimated lever contributions of the four joints to compute a measure of kinematic efficiency for each keystroke. Efficiency (η, *eta*) is defined as the ratio of the summed joint lever contributions (*estΔTIP_DIP_, estΔTIP_PIP_, estΔTIP_MCP_, estΔTIP_WRIST_*) and the sum of the absolute values of these four measures:
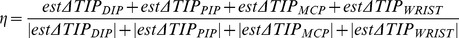



If all joints work together in the same direction to produce a keystroke, the efficiency measure will be 1.0. If some joints move in the opposite direction of each other, the efficiency will approach zero. This efficiency measure was computed for each individual keystroke; the mean values are shown by tempo condition for the group mean, S17 and S24 in Figure 9. A repeated-measures ANOVA conducted on the efficiency measures for the first five tempo conditions (in which all pianists participated) indicated no significant effect of tempo. As Figure 9 demonstrates, S17 shows reduced efficiency, and S24 shows increased efficiency of combined joint angle contributions to the downward fingertip trajectory during keystrokes, relative to the group mean. In addition, S24’s efficiency is improved across tempo conditions, whereas S17’s combined joint angle efficiency decreases at faster tempo performances.

### Correlations of Keystroke Efficiency with Acoustic Measures

Finally, we correlated the keystroke efficiency measures with the acoustic accuracy and precision of pianists’ performances. If joint angles contribute in an efficient manner to the arrival time and force goals of individual keystrokes, then high efficiency measures should correspond to increased precision and accuracy of timing and tone intensities (correlated with piano key velocity). The efficiency measures were correlated with the CV of inter-onset timing (SD/mean IOI), relative timing error, and CV of tone intensities (as defined earlier) across each melody cycle (*n* = 12) in each of 5 tempo conditions of the 12 pianists (*n* = 12 pianists × 5 tempi × 12 cycles = 720). Those correlations were all negative; as expected, combined joint efficiency increased as timing variability (CV of IOI) decreased (*r* = –.117, *p*<.005), as relative timing error decreased (*r* = –.066, *p* = .079), and as tone intensity variability (CV of MIDI key velocity) decreased (*r* = –.231, *p*<.001). The same correlations were computed at the level of individual pianists (*n* = 12); these correlations approached significance for the timing variability measures (*r* = –.540, *p* = .070) and reached significance for tone intensity CV’s (*r* = –.58, *p*<.05). These results suggest that the more efficient the combined joint movements of pianists’ fingers and wrist movements, the more accurate and precise the resulting tone onsets and more precise the tone intensities. This is the first measure we know of to connect kinematic efficiency with specific acoustic goals of performing musicians.

## Discussion

This study is the first to document the entire joint movement chain from skilled pianists’ forearms to fingertips (including all finger and hand joints) as they performed a five-finger task at increasingly fast tempi. We analyzed individual joint angles in an attempt to quantify each joint’s contribution to the final movement goal of pianists’ fingertips as they reached piano keys. Surprisingly, the individual joint contributions remained stable across tempo conditions; only the wrist movement contributed slightly more to the fingertip motion at fast tempi than at slow tempi. We also quantified movement efficiency for each individual finger keystrokes (keystroke efficiency) and found significant relationships with timing accuracy and precision of the produced sequences, suggesting that despite the many degrees of freedom a pianist may choose from, finger movements with larger keystroke efficiency are indeed beneficial for producing precisely and accurately timed sequences at fast rates. Other factors may also interact with performance timing, including anatomical, biomechanical, and physiological constraints; it does not follow that greater movement efficiency is always associated with better sequence timing. Furthermore, keystroke efficiency remained stable across a wide range of tempo conditions. This finding is consistent with results from a recent glove-based study on piano performance [17] that reported no difference in joint velocity covariation between striking and non-striking fingers at MCP and PIP joints across two tempi. This tempo invariance in the joint angle structure may be related to extensive practice of fundamental finger and hand movements in piano performance [17].

Large individual differences were documented in performance benchmarks of fastest achieved tempo condition, timing accuracy, and precision; the “fast” pianist produced faster and more accurately timed sequences than the “slow” pianist who deviated more from the prescribed tempo as it became faster. The “fast” pianist produced the keystroke movements from the knuckle (MCP) joint, whereas the other finger joints (PIP and DIP) extended only marginally. In contrast, the “slow” pianist extended the finger joints (PIP and DIP) considerably during a keystroke and had to compensate for this through exaggerated MCP flexion. In another comparison, the “fast” pianist showed higher than average efficiency measures (all joints worked in the direction of the finger’s movement goal toward the piano key), whereas the “slow” pianist exhibited lower than average efficiency measures (some joints moved opposite to the movement goal). These findings suggest that keystroke efficiency is a valid measure that is related to the temporal performance quality of the five-finger sequences. Whether or not anatomical and neuro-physiological properties contribute to these individual differences, these findings suggest that simple mechanical principles of movement efficiency may be influenced by individual motor optimization processes and vice versa [31].

These findings confirm recent educational recommendations that at medium to fast tempi, pianists’ finger technique is mainly accomplished by motion produced at the MCP joints (knuckles) with slight extension of the finger joints PIP and DIP. Consistent with this position, the piano educator Richard Beauchamp advised to play from the finger muscles and allow the finger “to unbend” passively (without active extension, nor flexion) [24], [25]. These observed movement patterns run against recommendations from other piano training schools to play with completely straight fingers, particularly for fast passages [30], or to employ a curved-finger stroke with a curled-in finger that extends to almost a straight finger during downswing [32]. It does not necessarily follow from our findings that other ways of striking the key are not advisable for piano performance; instead, the findings shown here indicate that when performing at fast tempi, pianists may be forced to use the observed movement patterns to accomplish the high temporal accuracy required of performance. Further investigations are necessary to generalize the keystroke efficiency findings reported here to other technical tasks in piano performance that require different degrees of difficulty (such as the well-learned movements required of scales and arpeggios).

Pianists’ finger movement patterns indicated a predominant use of the lumbrical muscles on the palmar side of the hand (intrinsic muscles of the hand), which bend the proximal finger phalanx while slightly extending the finger [18] rather than an extensive use of extrinsic finger muscles. Finger movements in piano performance differ from those of common tasks in motor control studies on finger dependence [19], [33] and, with practice, seem to be optimized for striking the piano keys with a specific timing and force. To achieve sufficient finger independence, pianists may avoid extensive use of the extrinsic muscles that are known to exhibit strong biomechanical coupling [34], [35] by generating movements from the lumbrical muscles that are known to be fairly independent [18]. However, more research is necessary to conclusively determine the use of intrinsic and extrinsic muscles in piano performance.

Skilled pianists who have spent thousands of hours on deliberate practice [1] may exhibit different, perhaps more efficient, neuro-structural organizations [36], which make them an ideal cohort for motor control research [37]. Finger movement patterns in expressive music performance have auditory outcomes as their ultimate goals. The more that all parts of the finger and wrist movement chain work jointly toward a movement goal (quantified here by keystroke efficiency), the higher the quality of the auditory outcome.

## Supporting Information

Video S1 **Pianists S17 and S24.** The video shows hand reconstructions of the smoothed marker data of Pianists S17 on the left and S24 on the right. The white spheres in the foreground (and one next to the pianists’ little finger) are markers attached to the piano keys. The original recordings were slowed down by a factor of 5.4 and 5.8, respectively (as S17 performed at a slightly slower rate than S24), to match each other visually. The video file does not contain sound.
(MP4)
